# Therapeutic stem cell‐derived alveolar‐like macrophages display bactericidal effects and resolve *Pseudomonas aeruginosa*‐induced lung injury

**DOI:** 10.1111/jcmm.17324

**Published:** 2022-04-20

**Authors:** Sheena Bouch, Michael L. Litvack, Kymberly Litman, Lisha Luo, Alex Post, Emma Williston, Amber J. Park, Elyse J. Roach, Alison M. Berezuk, Cezar M. Khursigara, Martin Post

**Affiliations:** ^1^ 7938 Translational Medicine The Hospital for Sick Children Toronto Ontario Canada; ^2^ Laboratory Medicine and Pathobiology The University of Toronto Toronto Ontario Canada; ^3^ Department of Molecular and Cellular Biology The University of Guelph Ontario Canada

**Keywords:** alveolar macrophage, antibiotic resistance, bacterial lung injury, bactericidal effects, pluripotent stem cell

## Abstract

Bacterial lung infections lead to greater than 4 million deaths per year with antibiotic treatments driving an increase in antibiotic resistance and a need to establish new therapeutic approaches. Recently, we have generated mouse and rat stem cell‐derived alveolar‐like macrophages (ALMs), which like primary alveolar macrophages (1'AMs), phagocytose bacteria and promote airway repair. Our aim was to further characterize ALMs and determine their bactericidal capabilities. The characterization of ALMs showed that they share known 1'AM cell surface markers, but unlike 1'AMs are highly proliferative *in vitro*. ALMs effectively phagocytose and kill laboratory strains of *P*. *aeruginosa* (*P*.*A*.), *E*. *coli* (*E*.*C*.) and *S. aureus*, and clinical strains of *P*.*A*. *In vivo*, ALMs remain viable, adapt additional features of native 1'AMs, but proliferation is reduced. Mouse ALMs phagocytose *P*.*A*. and *E*.*C*. and rat ALMs phagocytose and kill *P*.*A*. within the lung 24 h post‐instillation. In a pre‐clinical model of *P*.*A*.‐induced lung injury, rat ALM administration mitigated weight loss and resolved lung injury observed seven days post‐instillation. Collectively, ALMs attenuate pulmonary bacterial infections and promote airway repair. ALMs could be utilized as an alternative or adjuvant therapy where current treatments are ineffective against antibiotic‐resistant bacteria or to enhance routine antibiotic delivery.

## 1 INTRODUCTION

Communicable bacterial lung infections affect hundreds of millions of individuals, with respiratory infections being the leading cause of death in developing countries accounting for greater than 4 million deaths per year.^1^ Common bacterial infections and diseases include pneumonia, tuberculosis and pertussis; however, patients with compromised lung immunity, such as individuals with cystic fibrosis (CF), chronic obstructive pulmonary disease (COPD), bronchopulmonary dysplasia (BPD) and lung transplant recipients, are particularly susceptible to bacterial lung infections.^2^, ^3^, ^4^, ^5^ Furthermore, in light of the SARS‐CoV‐2 pandemic, in developed countries ~8% of critically ill SARS‐CoV‐2 patients present with bacterial co‐infections and antimicrobial therapies are routinely administered as a prophylactic measure,^6^, ^7^ potentially leading to a further increase in antibiotic resistance in our rapidly evolving healthcare landscape.^8^, ^9^ Recently, the World Health Organization published a list of 12 multi‐antibiotic‐resistant priority pathogens that pose the greatest threat to global human health; four of these directly colonize the lungs, namely *Pseudomonas aeruginosa* (*P*.*A*.), methicillin‐resistant *Staphylococcus aureus* (MRSA), *Streptococcus pneumonia,* and *Haemophilus influenzae*.^10^, ^11^ Current trends suggest that traditional antibiotic therapy is becoming less effective in clearing infections, resulting in longer hospital visits, higher medical costs and increased mortality.^8^ Thus, innovative approaches for new therapies need to be developed to combat antibiotic resistance. Cell‐based therapies hold promise as a revolutionary therapeutic to treat respiratory infections and halt global antibiotic resistance.

Primary alveolar macrophages (1'AMs) are tissue‐resident macrophages of the pulmonary innate immune system that maintain healthy airways by orchestrating the clearance pathogens and initiating an adaptive immune response.^12^, ^13^ Unlike bone marrow‐derived *Myb* ^+^ macrophages, which arise from adult hematopoietic stem cells, *Myb* ^−^ 1'AMs are from embryonic or foetal origin and self‐replicate, making them an ideal candidate for cell‐based therapies.^14^, ^15^, ^16^ In recent years, we have developed novel alveolar‐like macrophages (ALMs) that are derived from pluripotent stem cells (PSCs) and retain key embryonic signatures of *Myb* ^−^ 1'AMs.^17^ Previously, we have demonstrated that ALMs reduce lung injury in mouse models of acute and chronic lung diseases, can internalize dead *Staphylococcus aureus* (*S*.*A*.)^17^ and kill respiratory syncytial virus (RSV) and prevent RSV‐induced lung injury.^18^ Here, we further characterized ALMs for their efficacy to treat bacterial lung infections. We evaluated the non‐pharmacological antimicrobial functions of ALMs, specifically for bacteria known to colonize the lungs of immune‐compromised individuals, such as *P*.*A*. and *S*.*A*. in CF patients, and *Escherichia coli* (*E*.*C*.) that is nosocomially acquired in many pneumonia patients. Lastly, we also illustrate ALMs ability to resolve *P*.*A*.‐induced lung injury in a pre‐clinical model.

## 2 MATERIALS AND METHODS

### 2.1 Maintenance, expansion and culturing of alveolar‐like macrophages

ALMs were differentiated from mouse (G4 DsRed‐MST) and rat (DAc8) PSC lines and cultured in macrophage media as previously described.^17^ For bacterial experiments, penicillin‐streptomycin was removed 48 h before. ALMs were passaged every 5–7 days at a ratio of 1:10 using ReLeSR. Details of all reagents are reported in Table S1.

### 2.2 Collection of mouse and rat primary alveolar macrophages

Animal experiments were completed in compliance with the Animal Care Committee at The Hospital for Sick Children (AUP #46059 and #59356). Adult male C57BL6/J mice and Sprague‐Dawley rats were sacrificed with pentobarbitol. The thoracic cavity was opened and a tracheostomy was performed, then the lungs were instilled with Dulbecco's phosphate‐buffered saline (DPBS), and bronchoalveolar lavage fluid (BALF) was collected. For mice, lungs were lavaged with a 24‐gauge angiocath with 3 × 1 ml DPBS. For rats, lungs were lavaged with a 20‐gauge angiocath with 3 × 10 ml DPBS. Each aliquot was used to lavage the lungs twice. BALF collected was centrifuged at 400*g* for 20 min. The pellet was resuspended in macrophage media, transferred to tissue culture plates and incubated. Primary (1') AMs attached in 2 h and non‐adherent cells were removed.

### 2.3 *In vitro* proliferation of primary alveolar macrophages and alveolar‐like macrophages

1'AMs and ALMs were cultured at the same density in macrophage media. Cells were imaged using the same x/y coordinates at ×100 magnification on Days 0–7 using a light microscope (Leica DMI300B) and Volocity software (Perkin Elmer Inc, Version 6.3). The number of cells per field of view was manually counted.

### 2.4 Flow cytometry characterization of primary alveolar macrophages and alveolar‐like macrophages

Mouse ALMs were previously characterized for known 1'AM markers.^17^ Here, we further characterized mouse ALMs and 1'AMs for their expression of toll‐like receptor (TLR) 2, 4 and 5. Rat ALMs and 1'AMs were characterized using known 1'AM cell surface markers; CD45,^19^ SIRPα,^19^ CD11b/c^20^ and mature macrophage marker,^21^ along with TLR2, 4 and 5. Briefly, 1'AMs and ALMs were collected and the FcϒII receptor was blocked with anti‐CD32 for 5 min. All antibodies were added for 30 min. Further details are outlined in Table S2 or previously described.^17^ A Gallios 10/3 flow cytometer (Beckman‐Coulter) was used for acquisition and data were analysed using Kaluza software (Beckman‐Coulter).

### 2.5 Scanning electron microscopy of mouse alveolar‐like macrophage interactions with *P. aeruginosa*

Mouse ALM cultures were incubated in FluoroBrite DMEM containing 0.5% (v/v) FBS and carbenicillin at 300 μg/ml. Twenty million colony forming units (CFU) *P*.*A*. (PA01pMF230) containing a green fluorescent protein (GFP) plasmid (Addgene ID: Plasmid #62546, PA01pMF)^22^ were added for 20 min at 37°C. Cultures were then treated with 200 μg/ml gentamicin for 20 min at 37°C to kill planktonic bacteria. Cultures were washed and resuspended in FluoroBrite DMEM. Cultures were prepared for electron microscopy by washing with Na_2_HPO_4_: KH_2_PO_4_ buffer (1:1), fixing with 2% (v/v) glutaraldehyde and 1% (w/v) osmium tetroxide and dehydrating in an ascending sequence of ethanol washes. Samples were dried using a Denton DCP‐1 Critical Point Dryer (Denton Vacuum), and sputter‐coated using a Denton Desk V TSC. Images were acquired using a FEI Quanta FEG 250 scanning electron microscope using high vacuum mode.

### 2.6 *In vitro* internalization of *E. coli* and *P. aeruginosa* by mouse and rat alveolar‐like macrophagess

Mouse ALMs derived from DsRed‐expressing ESCs^17^, ^23^ and rat ALMs stained with CellTrace™ Far‐Red Cell Proliferation Kit were cultured with live GFP‐expressing *E*.*C*. (DSM 1103) (ATCC, #25922GFP) and *P*. *A*. (PA01pMF230) containing a GFP plasmid (Addgene ID: Plasmid #62546, pMF230), at an MOI of 100 bacteria per 1 ALM (100:1) for 45–90 min. ALMs were fixed with 4% (v/v) paraformaldehyde and counterstained with DAPI. Live cell imaging of mouse ALMs cultured with *P*.*A*. was completed using a Leica DMi8 microscope (Leica Microsystems Inc), connected to a Quorum Discovery Spinning Disk system (Quorum Technologies Inc), using Metamorph software (Molecular Devices). Image processing, including iterative restoration (confidence limit = 99%, 10 iteration limit = 10) was completed using Volocity. Live cell imaging of mouse ALMs cultured with *E*.*C*. and rat ALMs cultured *E*.*C*. and *P*.*A*., with was completed using a Leica CTRMIC 6000 confocal microscope as previously described.^17^

### 2.7 *In vitro* bactericidal capacity of rodent alveolar‐like macrophages

To perform a gentamicin protection assay (GPA), ALMs were suspended in Minimum Essential Media (MEM) with 0.5% (v/v) FBS and plated at 1.5 × 10^5^ ALMs in 50 µl per well of a 96‐well plate. Laboratory strains of *P*.*A*. (wild‐type), *E*.*C*. (ATCC, #25922), *S*.*A*. (Life Science Technologies, #S23371) and clinical respiratory strains of *P*.*A*. were grown in Lennox L Broth for 17 h on a bacterial shaker (220 rpm) at 37°C. Bacterial cultures were centrifuged at 4000*g* for 20 min and resuspended in DPBS, and cultures were analysed using a spectrophotometer (Beckman‐Coulter). CFUs were estimated by comparing OD_600_ values against a known OD_600_ value/CFU ratio. Bacteria were diluted in MEM with 0.5% (v/v) FBS at 7.5 × 10^5^ CFUs in 50 µl to achieve a MOI of 5:1. ALMs and bacteria were co‐incubated for 30 min at 37°C. Planktonic bacteria were killed by adding MEM containing 200 µg/ml gentamicin. The T0 and T2 plates were removed after 15 min and 2 h, respectively. ALMs were lysed with 1% (v/v) saponin and water. The lysate was centrifuged for 5 min at 6000*g* and resuspended in MEM containing 100 µg/ml of DNAse I on a shaker (1000 rpm) for 25 min at 37°C. Recovered bacteria were plated at dilutions of 10^0^–10^−2^ onto LB‐agar petri dishes and incubated overnight at 37°C. CFUs were counted the following day. Relative bacterial survival was calculated by normalizing the T0 absolute CFUs to 100% and expressing T2 absolute CFUs as a percentage of T0 (Killing Efficiency = ((# of T0 CFU‐# of T2 CFU)/# of T0 CFU) × 100%). Data were compiled from ≥3 biological replicates each having 3 technical replicates.

### 2.8 *In vivo* proliferation and viability of mouse and rat alveolar‐like macrophages

Mice and rats were anaesthetized with a loading dose of ketamine (mice 20 mg/kg, rat 75 mg/kg) and xylazine (mice 10 mg/kg, rat 25 mg/kg), followed by a 2 min exposure to 2% isofluorane. Four million mouse ALMs or 1 × 10^7^ rat ALMs were instilled intratracheally into the lungs via either a 24‐ or 20‐gauge angiocath. Mice ALMs expressed DsRed for tracking.^17^ Rat ALMs were prestained with CellTrace™ for tracking. Following 24 h, BALF was collected and the viability of ALMs was determined by flow cytometry using cell viability VivaFix assay. Proliferation of ALMs was determined by flow cytometry using an anti‐Ki67 antibody. Further antibody details are in Table S2. Flow cytometry results were gated using the DsRed and CellTrace™ dye fluorescence to distinguish the administered ALMs from primary BALF cells.

### 2.9 *In vivo* characterization of mouse and rat alveolar‐like macrophages and primary BALF cells

Mice and rats were anaesthetized and 4 × 10^6^ DsRed‐expressing ALMs or 10 × 10^6^ CellTrace™‐expressing ALMs, respectively, were instilled. Following 72 h, BALF was collected as described above. Characterization of *in vitro* ALMs, *in vivo* ALMs and primary BALF cells was determined by flow cytometry for known M1/M2 phenotypic markers CD80, CD86, CD206, Arg1, iNOS and Siglec‐F (mice only). Additionally, neutrophil influx post‐ALM instillation was determined by CD11b/c and GR‐1 expression in the primary BALF cell population. Further antibody details are in Table S2.

### 2.10 *In vivo* internalization and killing of *E. coli* and *P. aeruginosa* by rat and mouse alveolar‐like macrophages

Live GFP‐expressing *E*.*C*. and *P*.*A*., mouse DsRed ALMs and rat CellTrace™ ALMs were prepared as described above. For mice, 1 × 10^7^ bacteria were instilled while rats were instilled with 1 × 10^8^ bacteria. ALMs were instilled 30 min after the initial bacterial instillation to achieve a MOI of 10:1 for bacteria:ALMs (mice: 1 × 10^6^ ALMs, rats: 1 × 10^7^ ALMs).

### 2.11 Internalization of *E. coli* and *P. aeruginosa* by rat and mouse alveolar‐like macrophages

BALF was collected 3 h post‐instillation. Flow cytometry was performed on BALF, gating for GFP (bacteria) and DsRed or CellTrace™. Mouse GFP^+^DsRed^+^ and rat GFP^+^CellTrace™^+^ cells were considered ALMs that had internalized bacteria. Internalization was confirmed by creating cytospots and ensuring co‐localization of GFP and DsRed or CellTrace™ staining. For cytospots, 1 × 10^5^ ALMs were adhered to slides at 300*g* and fixed with 4% (v/v) PFA for 20 min. Mouse GFP^+^DsRed^+^ ALMs were imaged on a Leica DM 6000B microscope connected to a Leica TCS SP5 system using Leica Application Suite Advanced Fluorescence software (version 1.6.0) (Leica Microsystems Inc). GFP^+^CellTrace™^+^ ALMs were imaged using confocal microscopy and analysed as outlined above. Figure S1 provides a schematic experimental workflow.

### 2.12 Killing capacity and planktonic bacterial load in *P. aeruginosa*‐induced lung injury model in rats

To assess rat ALMs ability to kill *P*.*A*. over 24 h *in vivo* and determine the bacterial load, BALF was collected either 3 h (T0) or 27 h (T24) post‐*P*.*A*. instillation from rats that either received ALMs or a DPBS vehicle control. BALF was centrifuged at 400*g* for 10 min. BALF supernatant was collected and plated onto LB‐plates at dilutions of 10^0^–10^−8^. BALF cells were treated with MEM containing 200 µg/ml gentamicin for 30 min, centrifuged and resuspended in sort buffer for fluorescence‐activated cell sorting (FACS). FACS was performed on a MoFlo XDP Cell Sorter (Beckman‐Coulter). ALMs and primary BALF cells were separated based on CellTrace™ staining. A small subset of cells was used to create a cytospot for morphological analysis. A GPA was performed on the ALMs and primary BALF cells from the lysis step onwards as described above. Bacterial load (BALF supernatant) and survival (ALMs and primary BALF cells) were calculated as described above using the T0 and T24 time points. Figure S2 provides a schematic experimental workflow. This experiment was not performed in mice as a significant number of cells need to be recovered for FACS, therefore requiring a large number of mice to be used that could not ethically be justified.

### 2.13 Administration of alveolar‐like macrophages in *P. aeruginosa*‐induced lung injury model in rats

To determine the role of ALMs in *P*.*A*.‐induced lung injury, 1 × 10^8^ PA01 was intratracheally instilled into rats, with controls receiving DPBS. Six hours later, both groups were instilled with either 1 × 10^7^ ALMs or a DPBS control. Rats were weighed daily for seven days. At Day 7 the right lung was collected to determine the wet/dry weight ratio and the left lung was pressure fixed at 20 cm H_2_O with 4% (v/v) PFA and the tissue was processed through a number of ethanol, xylazine and wax baths before being embedded. Three 5 μm sections were cut 100 μm apart and stained with haematoxylin and eosin. From each section, 20 images were randomly taken in a grid‐like fashion with a ×200 magnification on a light microscope and Volocity as described above. An injury score was assigned to each image based on the presence and severity of epithelial thickening, epithelial sloughing, oedema and infiltrate. Each parameter was scored out of two with a final score out of eight. Three researchers independently scored 60 images per rat in a double‐blinded fashion, and an average score was taken per rat from all three researchers. Figure S3 provides a schematic experimental workflow.

### 2.14 Statistical analysis

Data are presented as mean ± SEM. Comparisons between groups were made using paired Student's *t*‐test using Welch's correction where appropriate. Where multiple comparisons were required, either a one‐way, two‐way or repeated‐measures ANOVA was used with either a Sidak or Tukey post hoc test. Statistical significance was set at *p* < 0.05. Statistical analysis and graphing were performed using GraphPad Prism 6.

## 3 RESULTS

### 3.1 Alveolar‐like macrophages proliferate *in vitro*

**FIGURE 1:**
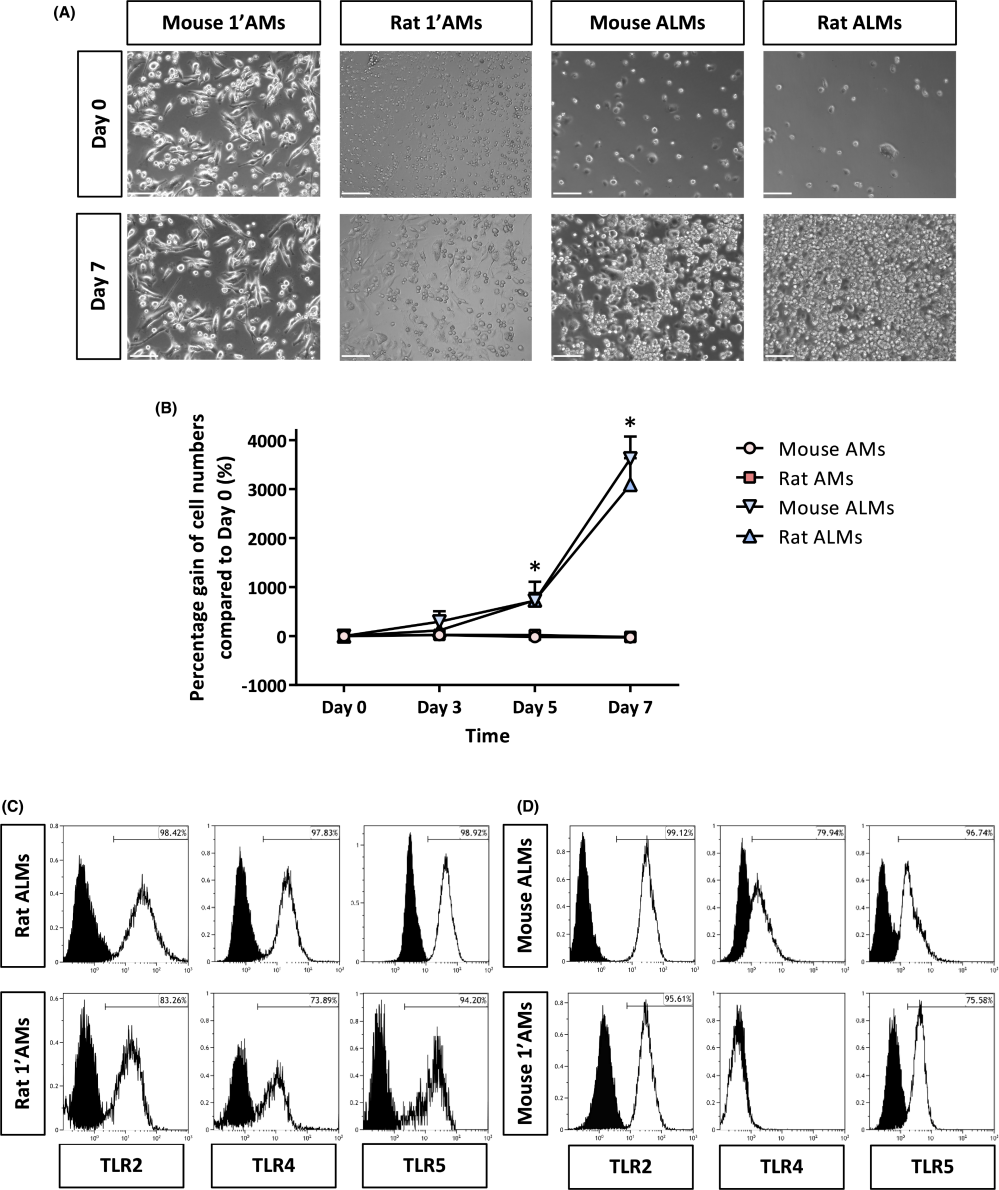
Characterization of rodent alveolar‐like macrophages. (A) Light micrographs representing the proliferation of mouse and rat primary isolated alveolar macrophages (1'AMs) and stem cell‐derived ALMs at Day 0 and Day 7 of culture. Images were taken at ×50 magnification. Scale bar is 150 μm. (B) Relative *in vitro* proliferation growth curves of mouse and rat ALMs compared with mouse and rat 1'AMs, respectively. Data are mean ± SEM, *n* = 3 separate experiments each with 3 technical triplicates. Statistically significant difference; * denotes *p* < 0.0001 between ALMs and 1'AMs. Data were expressed as a percentage gain in cell number to normalize for the variable attachment of cells observed in individual fields of view. (C) *In vitro* rat ALMs and 1'AMs express TLR2 and co‐express TLR4 and TLR5; (D) mouse *in vitro* ALMs express TLR2 and co‐express TLR4 and TLR5 whereas 1'AMs express TLR2 and TLR5 only. Black histograms; unstained control cells, white histograms; stained cells. All histograms are representative of *n* = 3–5 *in vitro* and *in vivo* experiments

The proliferative capabilities of ALMs and 1'AMs were directly compared. The doubling rate of rat and mice ALMs was 34.20 ± 1.25 and 46.40 ± 1.73 h, respectively, while 1'AMs did not divide, and the number of 1'AMs even decreased over seven days (Figure 1A and B). The experiment was terminated at seven days as the ALMs overgrow the culture dish at this point, but after passaging they continue to proliferate and can be continuously cultured *in vitro* for at least two years without a reduction in growth rate.^17^

### 3.2 Alveolar‐like macrophages express known primary alveolar macrophage cell surface markers

Rat ALMs and 1'AMs were compared for their surface marker expression (Figure S4A, *n* = 5). The rat ALM population highly expressed the 1'AM markers SIRPα, CD11b/c, mature macrophage marker, however, did not express the pan myeloid marker CD45, and CD86. Rat 1'AMs highly expressed CD45 and SIRPα, however, did not express the mature macrophage marker, CD11b/c or CD86. Mouse ALMs and 1'AMs expressed F4/80, CD11c, Siglec F, CD24, CD80, CD86 and CD206 but did not express MHCII or Langerin. CD64 was expressed on mouse ALMs but not on 1'AMs^17^ (Figure S4B, *n* = 5).

### 3.3 Alveolar‐like macrophages express TLR2, TLR4 and TLR5

ALMs and 1'AMs were analysed for surface markers TLR2, TLR4 and TLR5. Mouse ALMs and 1'AMs highly express TLR2 and co‐express TLR4 and TLR5 (Figure 1C, *n* = 3–5). Rat ALMs highly express TLR2 and co‐expresses TLR4 and TLR5 whereas the rat 1'AM population highly express TLR2 and TLR5 but not TLR4 (Figure 1D, *n* = 3–5).

### 3.4 Alveolar‐like macrophages internalize live *E. coli* and *P. aeruginosa*

**FIGURE 2:**
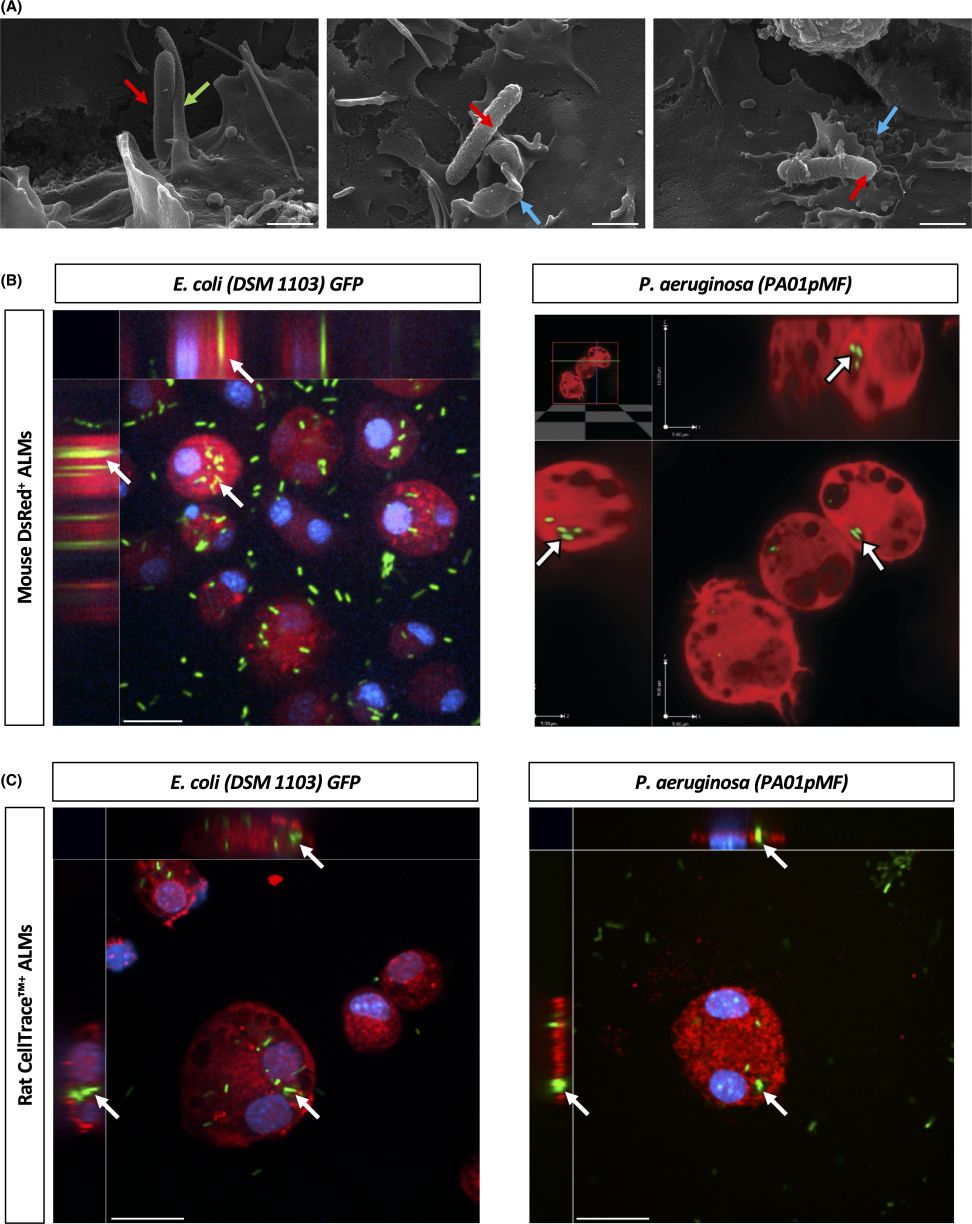
Alveolar‐like macrophages internalize live bacteria *in vitro*. (A) Scanning electron micrographs of mouse ALMs internalizing *P*.*A*. (PA01). ALM pseudopodia (green arrow) associate with a PA01 bacterium (red arrows) and PA01 bacterium is internalized by the phagocytic cup (blue arrows). Scale bars are 1 μm. (B) Confocal micrographs of DsRed‐expressing mouse ALMs (red) internalizing live GFP‐expressing *E*.*C*. (DSM 1103) and *P*.*A*. (PA01pMF) (green). *E*.*C*. image counterstained with DAPI (blue staining). A multi‐layered Z stack was performed to confirm internalization. White arrows indicate examples of internalized bacteria. Images were taken at ×200 magnification. Scale bars are 25 μm. Images are representative of two independent experiments. (C) Confocal micrographs of rat ALMs (stained with a CellTrace™ dye) internalizing live GFP‐expressing *E*.*C*. (DSM 1103) and *P*.*A*. (PA01pMF) (green). Images were counterstained with DAPI (blue staining). A multi‐layered Z stack was performed to confirm internalization. White arrows indicate internalized bacteria. Images were taken at ×200 magnification. Scale bars are 25 μm. Images are representative of two (or more) independent experiments

Scanning electron micrographs demonstrated *P*.*A*. associating with mouse ALMs (Figure 2A). Pseudopodia extend from the surface of the ALM and wrap around a *P*.*A*., followed by formation in the phagocytic cup before beginning to envelope a *P*.*A*. Confocal imaging confirmed that mouse ALMs (Figure 2B) and rat ALMs (Figure 2C) internalize live *E*.*C*. and *P*.*A* and retain them in vacuolar structures resembling phagosomes.

### 3.5 Alveolar‐like macrophages display bactericidal effects to live *E. coli*, *P. aeruginosa* and *S. aureus*

**FIGURE 3:**
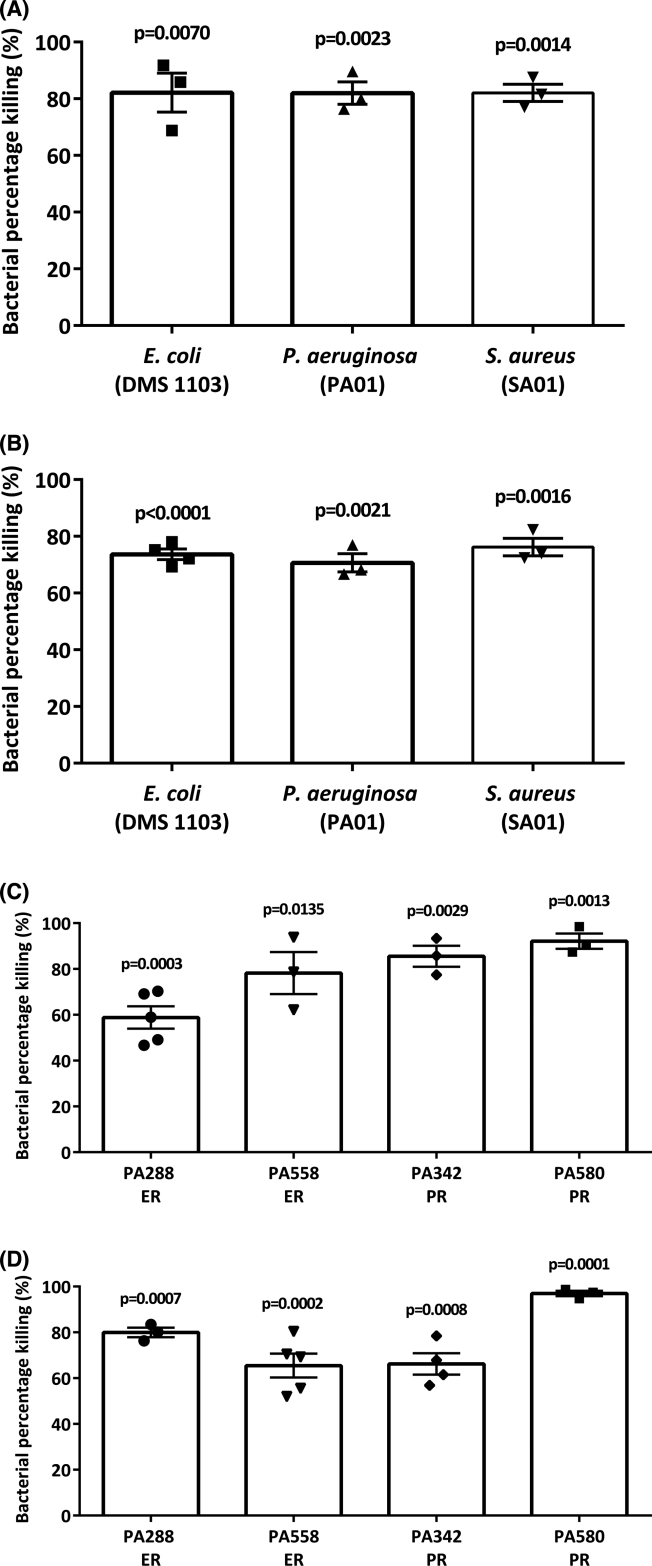
Bactericidal capacity of alveolar‐like macrophages to kill *P*. *aeruginosa*, *E*. *coli*, *S*. *aureus* and clinical respiratory strains of *P*. *aeruginosa in vitro*. Bacterial percentage killing for mouse (A) and rat (B) ALMs targeting live laboratory strains of *E*.*C*., *P*.*A*. and *S*.*A*. were determined using a GPA. Bacterial per cent killing for mouse (C) and rat (D) ALMs targeting various clinical *P*.*A*. strains that are either tobramycin sensitive and are eradicated by antibiotics (ER) or are resistant to tobramycin and persist with antibiotic treatment (PR) were determined using a GPA. Data in all panels are presented as mean ± SEM, *n* = 3–5 separate experiments each with technical triplicates. Statistical comparisons were made against the T0 time point (data not shown)

We evaluated the killing capacity of mouse ALMs in response to exposure to live *E*.*C*., *P*.*A*. and *S*.*A*. and observed killing capacities of 82.17 ± 6.90% (*p* = 0.0070, *n* = 3), 81.98 ± 3.95% (*p* = 0.0023, *n* = 3) and 82.05 ± 3.04% (*p* = 0.0014, *n* = 3), respectively, within 2 h of bacterial exposure (Figure 3A). The killing capacity of rat ALMs in response to exposure to live *E*.*C*., *P*.*A*. and *S*.*A*. was 73.66 ± 1.83% (*p* = <0.0001, *n* = 4), 70.64 ± 3.21% (*p* = 0.0021, *n* = 3) and 76.23 ± 3.08% (*p* = 0.0016, *n* = 3), respectively, within 2 h of bacterial exposure (Figure 3B). Re‐interpretation of this data illustrated that mouse and rat ALMs display similar bactericidal efficiencies to *P*.*A*. (Figure S5A).

### 3.6 Alveolar‐like macrophages display bactericidal effects to live clinical patient‐derived *P. aeruginosa* strains

Ten strains of *P*.*A*. were obtained from clinical isolates of CF patient sputum samples whose eradication status towards tobramycin treatment had been previously determined (Figure S5B). ALMs displayed bactericidal activity to all *P*.*A*. patient strains (representative examples in Figure 3C and D). The killing capacity of mouse ALMs was significantly increased (from 0% at T0) in response to the eradicated strains PA288 (58.82 ± 4.89%, *p* = 0.0003, *n* = 5) and PA558 (78.17 ± 9.17%, *p* = 0.0135, *n* = 3) and the persistent strains PA342 (85.54 ± 4.59%, *p* = 0.0029, *n* = 3) and PA580 (92.14 ± 3.31%, *p* = 0.0013, *n* = 3). The killing capacity of rat ALMs was significantly increased (from 0% at T0) in response to the eradicated strains PA288 (79.94 ± 2.07%, *p* = 0.0007, *n* = 3) and PA558 (65.49 ± 5.20%, *p* = 0.0002, *n* = 5) and the persistent strains PA342 (66.20 ± 4.67%, *p* = 0.0008, *n* = 5) and PA580 (96.96 ± 1.19%, *p* = 0.0001, *n* = 5). Re‐interpretation of this data illustrated that mouse and rat ALMs display similar bactericidal efficiencies to both laboratory and clinical strains of *P*.*A*. (Figure S5C).

### 3.7 Alveolar‐like macrophages are viable and proliferate *in vivo*

**FIGURE 4:**
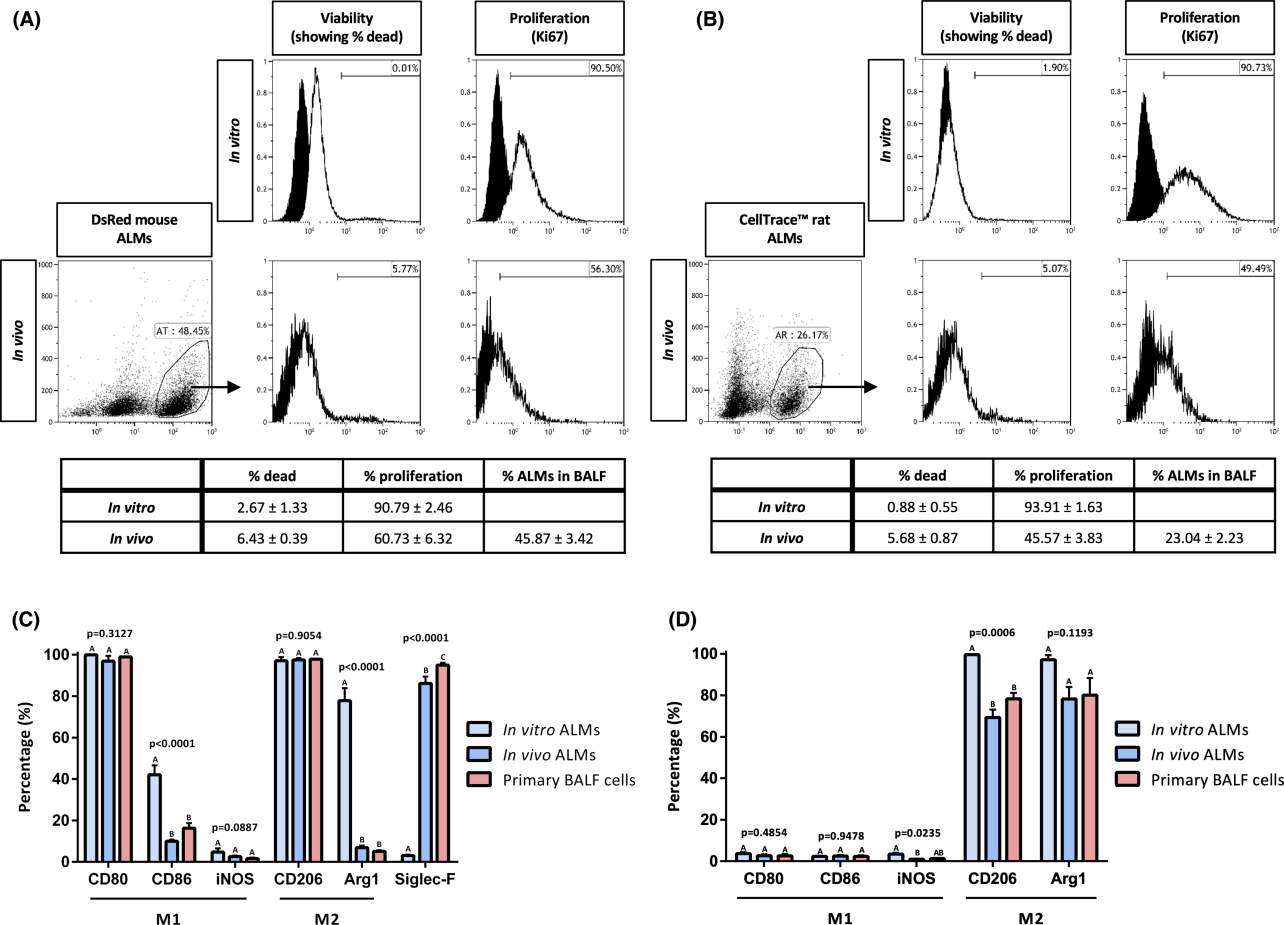
Alveolar‐like macrophages are viable but have reduced proliferative capacity *in vivo*. Mouse DsRed^+^ ALMs (A) and rat CellTrace™^+^ ALMs (B) are viable and proliferative, when cultured to 50% confluency *in vitro* prior to instillation. 24 h post *in vivo* instillation ALMs were collected via bronchoalveolar lavage fluid (BALF). The mouse DsRed^+^ ALM or rat CellTrace™^+^ ALM population in the BALF was separated from endogenous populations by flow cytometry. Mouse and rat ALMs retain their viability, but their proliferative capacity is diminished. Data in both panels are presented as mean ± SEM. Black histograms; unstained control cells, white histograms; stained cells. (A) and (B) histograms represent one of *n* = 3 for *in vitro* cultures and *n* = 4 separate *in vivo* ALM instillations. (C) Flow cytometry of mouse *in vitro* ALMs, and ALMs and primary BALF cells three days post‐instillation in mice for known M1/M2 polarization markers and siglec‐F. (D) Flow cytometry of rat *in vitro* ALMs, and ALMs and primary BALF cells three days post‐instillation in rats for known M1/M2 polarization markers. (C) and (D) Data are presented as mean ± SEM, *n* = 3–6 per experimental group

Twenty‐four hours post‐instillation, mouse ALMs retained 93.57 ± 0.39% (*n* = 3) and 60.73 ± 6.32% (*n* = 3), respectively, of their viability and proliferative capacity compared with 97.33 ± 1.33% (*n* = 3) viability and 90.79 ± 2.46% (*n* = 3) proliferative capacity prior to instillation (Figure 4A). Rat ALMs retained 94.32 ± 0.87% (*n* = 3) and 45.57 ± 3.83% (*n* = 3), respectively, of their viability and proliferative capacity compared with 99.12 ± 0.55% (*n* = 3) viability and 93.91 ± 1.63% (*n* = 3) proliferative capacity prior to instillation (Figure 4B).

### 3.8 Alveolar‐like macrophages adapt to the lung microenvironment *in vivo*

In mice and rats, three days post‐ALM administration the expression of M1/M2 markers was assessed in transplanted mouse DsRed^+^ ALMs, rat CellTrace™^+^ ALMs and primary BALF cells in relation to *in vitro* cultured ALMs. In mice, no difference was observed in expression of M1 markers CD80 (*p* = 0.3127, *n* = 3–6) and iNOS (*p* = 0.0887, *n* = 3–6), or M2 marker CD206 (*p* = 0.9054, *n* = 3–6) between the groups. However, expression of M1 marker CD86 and M2 marker Arg1 were markedly decreased in the transplanted ALMs and primary BALF cells compared to the *in vitro* ALMs, *p* < 0.0001, *n* = 3–6; *p* < 0.0001, *n* = 3–6, respectively (Figure 4C). Siglec‐F expression was significantly increased in transplanted ALMs and primary BALF cells compared to *in vitro* ALMs (*p* < 0.001, *n* = 3–4) (Figure 4C). Neutrophil influx (CD11b^+^/GR‐1^+^ population in the primary BALF cells) was marginally increased following instillation of ALMs (1.54 ± 0.27% vs 7.90 ± 1.30%, control vs. ALMs, *n* = 3–4). In rats, no difference was observed in the M1 markers CD80 (*p* = 0.4854, *n* = 3–4) or CD86 (*p* = 0.9478, *n* = 3–4) or the M2 marker Arg1 (*p* = 0.1193, *n* = 3–4) between the groups. Expression of the M1 marker iNOS was significantly decreased in the transplanted ALMs compared with the *in vitro* ALMs (*p* = 0.0235, *n* = 3–4) and the M2 marker CD206 was decreased in the transplanted ALMs and primary BALF cells compared to *in vitro* ALMs (*p* = 0.0006, *n* = 3–4) (Figure 4D). Neutrophil influx (CD11b/c^+^/GR‐1^+^ population in the primary BALF cells) was not significantly altered following instillation of rat ALMs (3.08 ± 0.49% vs 5.29 ± 1.53%, control vs. ALMs, *n* = 3–4).

### 3.9 Alveolar‐like macrophages internalize and kill bacteria *in vivo* without altering bacterial load in BALF

**FIGURE 5:**
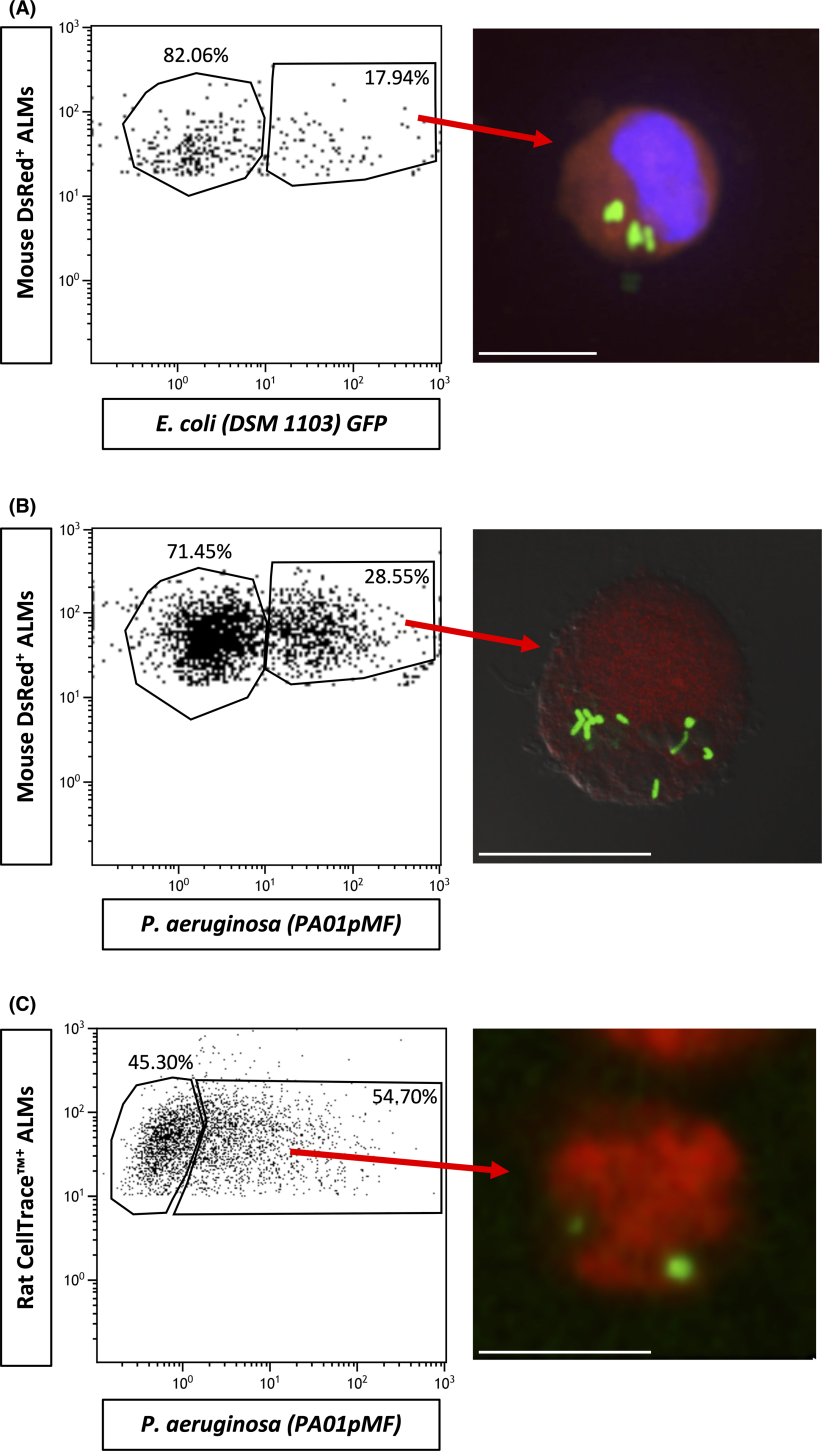
Alveolar‐like macrophages display bactericidal effects *in vivo* by internalizing *P*. *aeruginosa* and *E*. *coli*. Adult mice and rats were infected intratracheally with either live GFP‐expressing *E*.*C*. and/or *P*.*A*. Thirty minutes post‐infection, mice and rats were instilled intratracheally with mouse DsRed^+^ ALMs or rat CellTrace™^+^ ALMs, respectively. Three hours following the initial infection mice and rats were sacrificed and BALF cells were collected. (A) Flow cytometry on DsRed^+^/GFP^+^ of BALF cells from mice instilled with GFP‐expressing *E*.*C*. (B) Flow cytometry on DsRed^+^/GFP^+^ of BALF cells from mice infected with GFP‐expressing *P*.*A*. (C) Flow cytometry on CellTrace™^+^/GFP^+^ of BALF cells from rats infected with GFP‐expressing *P*.*A*. All flow cytometry gating strategies were performed on ALM only controls (data not shown). Respective corresponding confocal micrographs show composite confocal images with GFP bacteria within the red ALMs. Mouse DsRed^+^ ALMs + GFP‐expressing *E*.*C*. was counterstained with DAPI. All images were taken at ×200 magnification. Scale bars are 10 μm (A, B and C). Black histograms; unstained control cells, white histograms; stained cells (A), (B) and (C) histograms represent one of *n* = 3–4 separate *in vivo* ALM instillations

**FIGURE 6:**
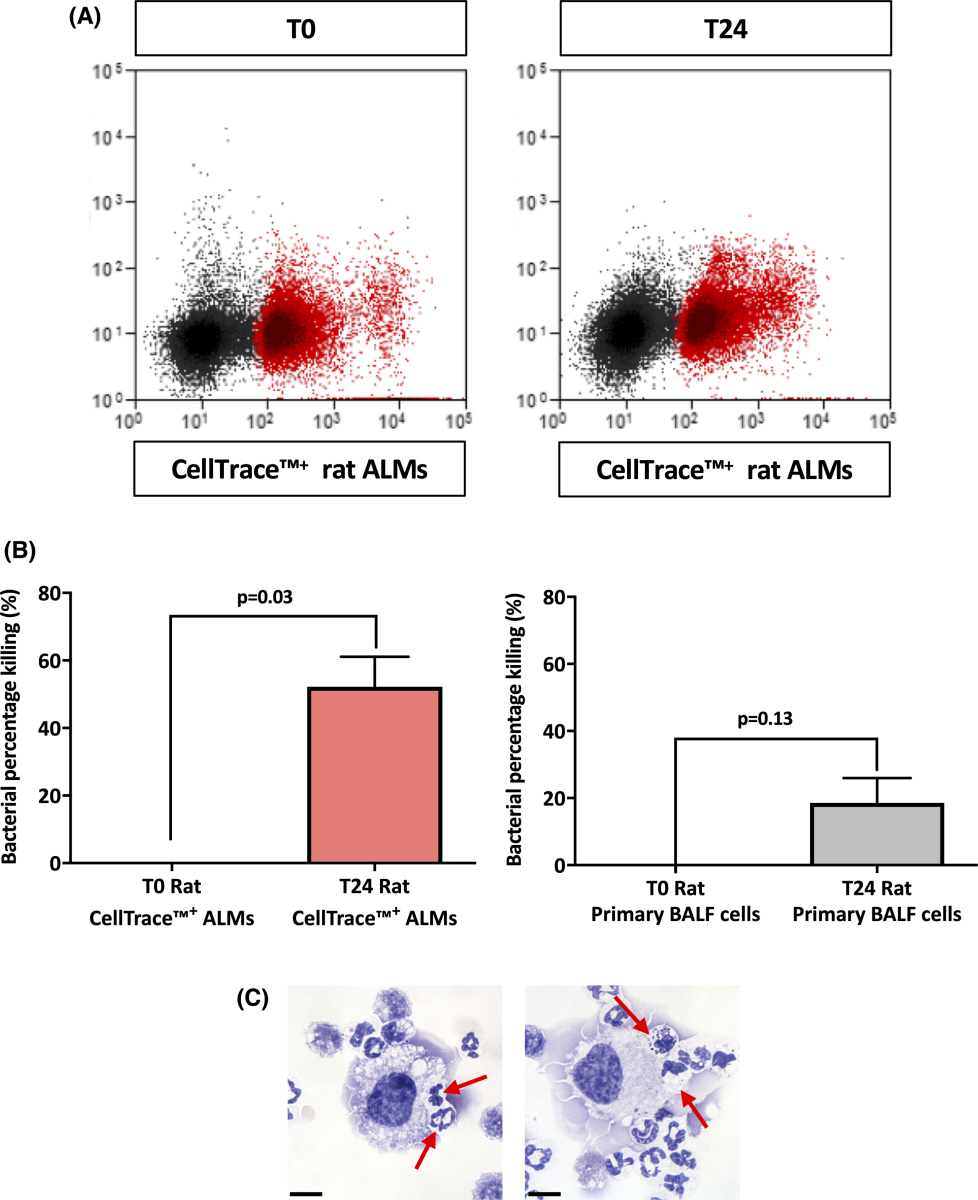
Alveolar‐like macrophages display bactericidal effects *in vivo* towards *P*. *aeruginosa*. Adult rats were infected intratracheally with GFP‐expressing *P*.*A*. Thirty minutes post‐instillation, rats were instilled intratracheally with CellTrace™ labelled ALMs or DPBS. Either 3 h (T0) or 27 h (T24) following instillation BALF was collected and cells were treated with gentamycin. (A) BALF cells collected at T0 and T24 were separated by FACS into CellTrace™^+^ ALMs or CellTrace™^−^ primary BALF cells (CellTrace™^+^ ALMs highlighted as red on scatter plot). (B) A GPA was performed on CellTrace™^+^ ALMs (red bar) and CellTrace™^−^ primary BALF cells (grey bar) demonstrating that CellTrace™^+^ ALMs were effectively killing *P*.*A*. *in vivo* over 24 h. Data are presented as mean ± SEM, *n* = 3 separate *in vivo* ALM instillations. (C) Light micrographs of ALMs associating with neutrophils post‐*P*.*A*. infection. Phagocytosis is indicated by the red arrows. All images were taken at ×670 magnification. Scale bars are 10 μm

Firstly, we established that mouse ALMs could internalize *E*.*C*. and *P*.*A*. *in vivo* over 3 h; 15.88 ± 4.48% and 31.83 ± 3.54% (*n* = 3) of mouse ALMs internalize *E*.*C*. and *P*.*A*., respectively (Figure 5A and B). Also 57.74 ± 3.71% (*n* = 3) of rat ALMs internalize *P*.*A*. (Figure 5C). Next, we assessed the ability of rat ALMs and primary BALF cells to kill *P*.*A*. over 24 h and reduce the bacterial load in BALF. Rat ALMs and primary BALF cells were separated at 3 [T0] and 27 h [T24] (Figure 6A) post‐instillation and a GPA was performed. The killing capacity of ALMs at T24 was significant from 0% at T0 to 52.20 ± 8.91% at T24, *p* = 0.03, *n* = 3 (Figure 6B), whereas the killing capacity of primary BALF cells at T24 was not significant from 0% at T0 to 18.54 ± 7.43% at T24, *p* = 0.13, *n* = 3 (Figure 6B). Of note, ALMs represented only 3.87 ± 0.60% (*n* = 6) of cells recovered in the BALF. Although the ALMs had a greater killing capacity, the bacterial load of BALF in the PA01 + DPBS group was not significantly different to the PA01 + ALM group (43.93 ± 16.60% vs 65.33 ± 7.143%, respectively, *p* = 0.33, *n* = 3). Interestingly, ALMs phagocytosed neutrophils *in vivo* (Figure 6C).

### 3.10 Rat alveolar macrophages resolve *P. aeruginosa*‐induced lung injury *in vivo*

**FIGURE 7:**
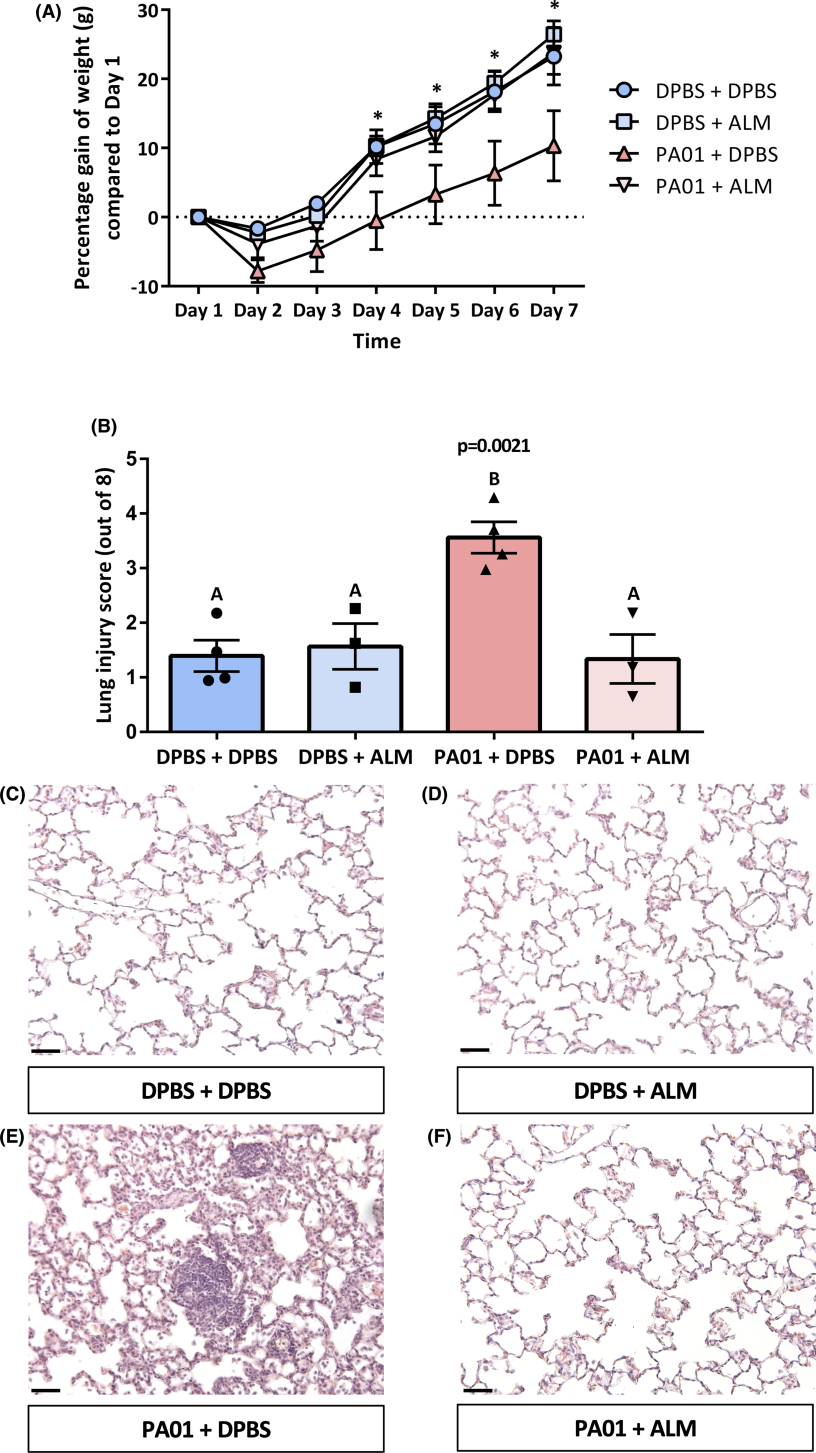
Alveolar‐like macrophages resolve *P*. *aeruginosa*‐induced lung injury *in vivo*. Adult rats were infected intratracheally with *P*.*A*. or DPBS. Six hours post‐instillation, rats were instilled intratracheally with either ALMs or DPBS. Rats were weighed daily, and histological analysis was performed at Day 7 post‐instillation. (A) Rats in the PA01 + DPBS group was lighter than the DPBS + DPBS and DPBS + ALM groups by Day 4 and all three experimental groups by Day 5 onwards. Data are mean ± SEM, *n* = 3–4 rats per experimental group. Statistically significant difference; * denotes *p* < 0.05 between experimental groups in a Tukey's post hoc test. Data were expressed as a percentage gain in body weight to normalize for the variable starting weights (pre‐instillation). (B) Lung histological analysis demonstrated that the injury score was significantly greater in the PA01 + DPBS group compared to the other three experimental groups, with no differences observed between the other groups. Data are mean ± SEM, *n* = 3–4 rats per experimental group. Experimental groups with different letters (above the bar) are significantly different from each other. (C–F) Representative histological images of DPBS + DPBS, DPBS + ALM, PA01 + DPBS and PA01 + ALM groups, respectively. Images were taken at ×200 magnification. Scale bars are 50 μm. Images are representative of 3–4 rats per experimental group

Body weight was measured daily for seven days post‐instillation. Differences were observed between groups (*p* ^group^ = 0.0645, *p* ^time^ < 0.0001, *p* ^interaction^ = 0.0028, *n* = 3–4 per group). Tukey's post hoc revealed the PA01 + DPBS group was lighter in weight than the DPBS + DPBS and DPBS + ALM groups by Day 4 and all three groups by Day 5 onwards (Figure 7A). No differences were observed for the wet/dry lung weights (data not shown). The morphometry injury score at Day 7 was significantly greater in the PA01 + DPBS group compared with the other three groups, with no differences observed between the other groups (*p* = 0.0021, *n* = 3–4; DPBS + DPBS 1.39 ± 0.29, DPBS + ALM 1.57 ± 0.42, PA01 + DPBS 3.56 ± 0.29, PA01 + ALM 1.34 ± 0.45) (Figure 7B–F). Further investigation attributed this greater injury score to an increase in epithelial thickening (*p* = 0.0086), epithelial sloughing (*p* = 0.0006) and infiltrate (*p* = 0.0141) but not oedema (*p* = 0.4468) in the PA01 + DPBS group (Figure S6).

## 4 DISCUSSION

Recently, the need to establish more effective therapeutic approaches towards lung diseases and infections has driven the development of cell‐based therapies.^24^, ^25^ This rapidly evolving area led to the development of ALMs,^17^ and here we have demonstrated that ALMs display potent bactericidal functions, contribute to the clearance of laboratory and patient‐derived strains of common bacteria and resolve *P*.*A*.‐induced lung injury.

Surface expression of known 1'AM markers^20^, ^21^, ^26^ was confirmed on rat ALMs establishing their similarity to 1'AMs. Rat ALMs similarly express CD86 and SIRPα; however, CD11b/c and the mature macrophage marker are highly expressed in rat ALMs compared to 1'AMs.^17^ This differential expression could be due to variable activation states of *in vitro* ALMs and *in vivo* 1'AMs, which is known to alter marker expression.^20^ CD45 is expressed by virtually all hematopoietic cells; however, some primitive macrophage subsets, such as microglia and those derived from yolk‐sac haematopoiesis express low or no CD45.^27^ Interestingly, mouse ALMs express CD45 but rat ALMs do not, suggesting that the expression of CD45 on ALMs is non‐essential to their characteristics and function in general and re‐affirming that ALMs retain a primitive signature.^17^, ^28^ Importantly, mouse and rat ALMs express TLRs 2, 4 and 5, known to be responsive to the pathogen‐associated molecular patterns of LPS and flagellin^29^, ^30^; and ALMs proliferate more sustainably and rapidly than 1'AMs and primary bone marrow‐derived macrophages.^17^ Mouse 1'AMs have been cultured to 20 passages *in vitro*,^15^, ^31^ and human iPSC‐derived macrophages have been harvested for up to three months post‐differentiation in a bioreactor system^32^; however, the rate of proliferation of our ESC‐derived ALMs is significantly greater and they remain proliferative over longer periods of time.^17^ ALMs are therefore easily scalable to therapeutic numbers, which is key to producing a cost and time effective cell‐based therapy.

One of the major effector functions of 1'AMs is their ability to phagocytose and kill pathogens.^33^ Thus, we determined the bactericidal effects of ALMs. After visualizing bacterial internalization, we confirmed that both rat and mouse ALMs effectively kill laboratory strains of *E*.*C*., *P*.*A*. and *S*.*A*. with greater than 70% killing efficiency. Moreover, ALMs efficiently kill clinical strains of *P*.*A*. It is important to recognize that ALMs effectively killed both eradicated and persistent *P*.*A*. clinical strains with the same efficiency as the laboratory strain. We speculate that ALMs will kill other strains of antibiotic‐resistant bacteria where traditional antibiotics are no longer effective. This finding has potentially valuable applications for therapeutics whereby lower concentrations of antibiotics could be used as a combinatorial strategy with ALMs to lessen the risk of developing antibiotic resistance, but still retain therapeutic bacterial eradication.

In the present study, we also characterized ALMs *in vivo* and demonstrated that ALMs remain viable (~95%) but drop their proliferation rate by ~50%, suggesting ALMs adapt to the lung niche and present as 1'AMs, which exhibit slow proliferation rates *in vivo*.^34^ This reduction in proliferation could be beneficial, as it suggests ALMs are less likely to become tumorigenic, an important distinction as ALMs can be retained in the airways for at least 4 weeks.^17^ In mice, ALM polarization towards a M1 or M2 phenotype was not observed; however, we observed that ALMs adapt to the lung niche by reducing their expression of CD86 and CD206, and increasing their expression of Siglec‐F to adopt a phenotype more analogous to 1'AMs. We also observed a marginal increase in the CD11b^+^/GR‐1^+^ neutrophil population in the BALF following ALM administration in mice. This increase from ~2% to ~8% is unlikely to indicate an acute inflammatory response as >90% neutrophils are observed in the BALF of inflammatory LPS models.^35^, ^36^ Unlike mice, rat ALMs exhibit a distinctly M2 phenotype, both *in vitro* and three days post‐instillation in rat lungs. After transplantation, rat ALMs also adopt a phenotype similar to 1'AMs, as seen with a reduction in CD206 expression to 1'AM levels.

Following *in vivo* characterization of ALMs, we demonstrated that mouse and rat ALMs internalized bacteria within 3 h post‐instillation. Additionally, during the 24 h following internalization rat ALMs effectively killed ~50% of internalized *P*.*A*., whereas primary BALF cells killed ~20% of internalized *P*.*A*., which is a notable difference in killing efficiency. However, ALMs only accounted for ~4% of cells present in the BALF and provides a likely explanation as to why ALM administration did not result in a reduction the overall bacterial load in the BALF.

Finally, we determined if ALM administration could resolve *P*.*A*.‐induced lung injury. Administration of ALMs ameliorated the loss of growth observed in the PA01 + DBPS group, which was evident from Day 4 onwards. This is an important finding as weight loss is a hallmark symptom of *P*.*A*.‐induced pneumonia.^37^, ^38^, ^39^ At Day 7, administration of ALMs mitigated *P*.*A*.‐induced structural lung injury, namely epithelial thickening, sloughing and infiltration (Figure 7B). One discrepancy is that despite a resolution of injury at Day 7 and mitigation of weight loss following ALM administration, there is no change in the bacterial load of BALF 24 h post‐infection. A limitation is that we only determined the bacterial load at 24 h post‐infection; however, it is plausible that the bacterial load may be reduced or resolved at a later time point, especially as the amelioration of weight loss was observed from Day 4 onwards. However, another mechanism for the ALM‐driven resolution of lung injury could be rat ALMs maintaining a predominant M2 phenotype within the lung microenvironment. It is probable that M2 ALMs can downregulate the magnitude and longevity of neutrophil influx and aid in efferocytosis, a known M2 macrophage effector function,^40^ resulting in the mitigation of *P*.*A*.‐induced structural lung injury and weight loss by Day 7. Future studies will be more expansive, including determining the bacterial load and cytokine profiles at different time points and whether greater ALM dosages can reduce the bacterial load even after 24 h.

Together, these data are the first to show ALM‐driven resolution of *P*.*A*.‐induced lung injury, and further supports the concept of ALMs promoting airway repair in disease models.^17^ Recently, it has been reported that murine hematopoietic stem cell transplantation^39^ or delivery of human iPSC‐derived macrophages^32^ rescued mice from *P*.*A*. airway infection; however, the critical distinction is that these graft‐derived or instilled macrophages were of bone marrow or non‐primitive origins, unlike ALMs that are engineered to reside in the lung for long periods of time. Moreover, in these studies the graft‐derived macrophages already resided in the lung prior to infection^39^ while human iPSC‐derived macrophages were instilled at the time of infection.^32^ In contrast, in our study ALMs were delivered post‐infection, which more realistically mimics a clinical time‐course.

## 5 CONCLUSIONS

Collectively, the use of ALMs as a translational cell‐based bactericidal therapy holds great promise in future. ALMs are a robust proliferative cell with the potential to be utilized as an alternative or adjuvant therapy where current strategies are ineffective against antibiotic‐resistant bacteria or to enhance routine antibiotic delivery. Importantly, ALMs not only resolve lung injury but also are effective antibacterial cells that attenuate pulmonary bacterial infection and promote repair. Moreover, ALMs scalability and immunological functionality make them versatile cells that are permissible to transfection and thus could be altered to further enhance their bactericidal and immunoregulatory functions and be used as a platform to treat a multitude of lung diseases.

## CONFLICT OF INTEREST

The authors confirm that there are no conflicts of interest.

## AUTHOR CONTRIBUTIONS

**Sheena Bouch:**Conceptualization (equal); data curation (lead); formal analysis (lead); funding acquisition (supporting); investigation (equal); methodology (equal); project administration (equal); writing – original draft (lead); writing – review & editing (equal). **Michael L. Litvack:** Conceptualization (equal); data curation (equal); formal analysis (equal); funding acquisition (supporting); investigation (equal); methodology (equal); project administration (equal); writing – original draft (supporting); writing – review & editing (equal). **Kymberly Litman:** Data curation (supporting); formal analysis (supporting). **Lisha Luo:** Data curation (supporting); formal analysis (supporting). **Alex Post:** Data curation (supporting); formal analysis (supporting). **Emma Williston:** Data curation (supporting); formal analysis (supporting). **Amber J. Park:** Data curation (supporting); formal analysis (supporting). **Elyse J. Roach:** Data curation (supporting); formal analysis (supporting). **Alison M. Berezuk:** Data curation (supporting); formal analysis (supporting). **Cezar M. Khursigara:** Data curation (supporting); formal analysis (supporting); supervision (supporting). **Martin Post:** Conceptualization (equal); formal analysis (supporting); funding acquisition (lead); investigation (equal); methodology (equal); project administration (equal); resources (equal); supervision (lead); writing – review & editing (equal).

## Supporting information

Fig S1

Fig S2

Fig S3

Fig S4

Fig S5

Fig S6

Table S1

Table S2

Figure Lagends

## Data Availability

The data that support the findings of this study are available from the corresponding author upon reasonable request.
