# Supplementary figures and images for: Therapeutic stem cell‐derived alveolar‐like macrophages display bactericidal effects and resolve Pseudomonas aeruginosa‐induced lung injury
Source: J Cell Mol Med. 2022 Apr 20;26(10):3046–59. doi: 10.1111/jcmm.17324 (PMC9097833; doi:10.1111/jcmm.17324)

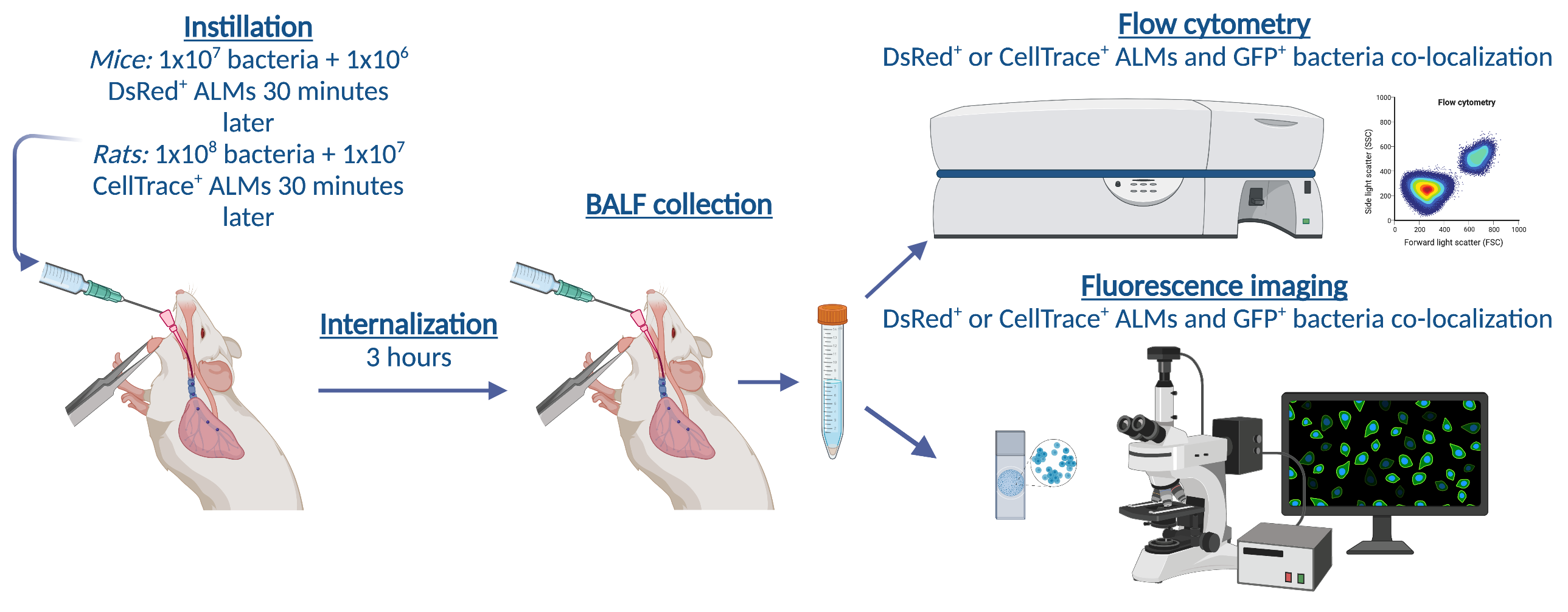

Supplement: Supplementary file 1 — Fig S1 [file JCMM-26-3046-s002.png]

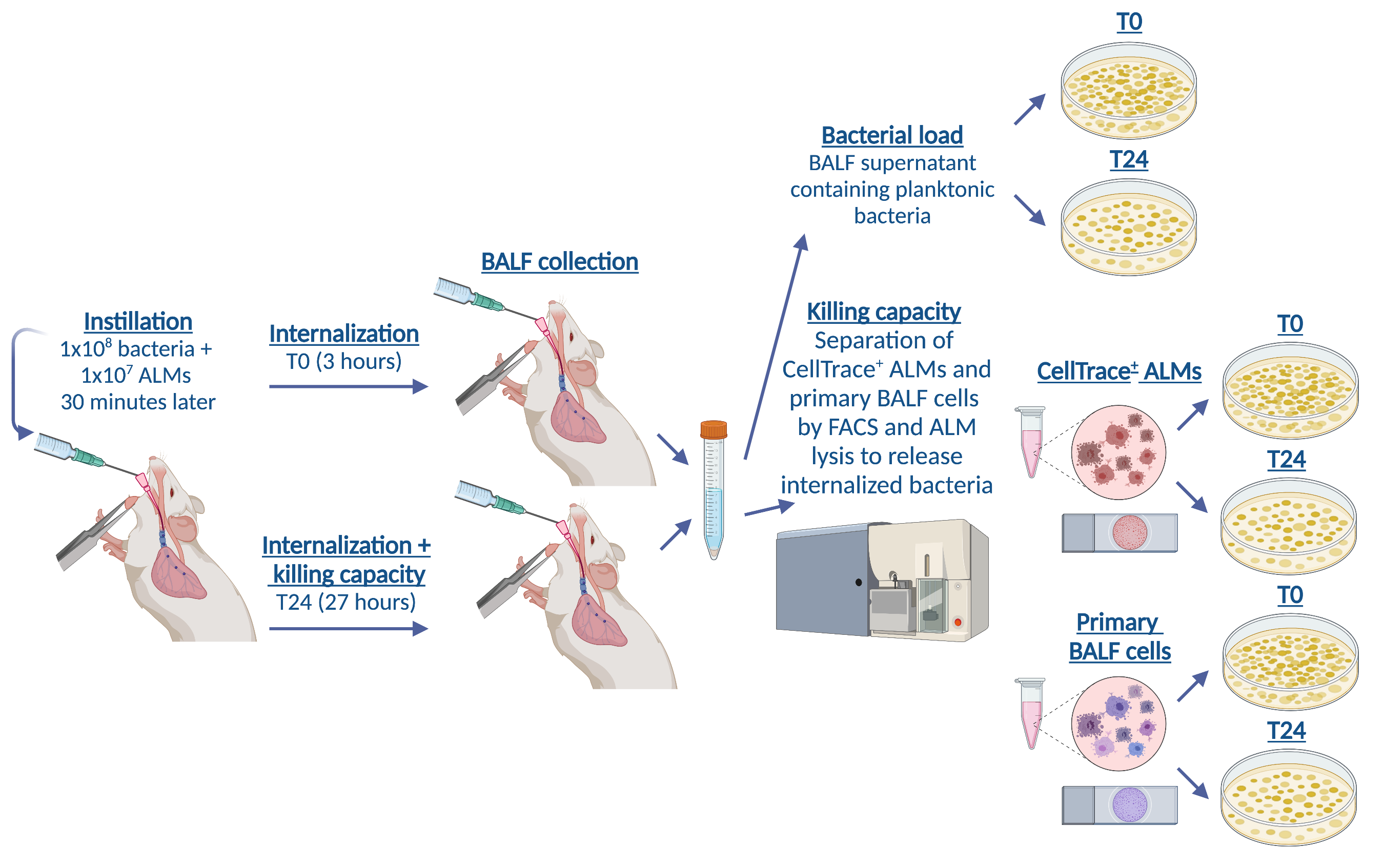

Supplement: Supplementary file 2 — Fig S2 [file JCMM-26-3046-s007.png]

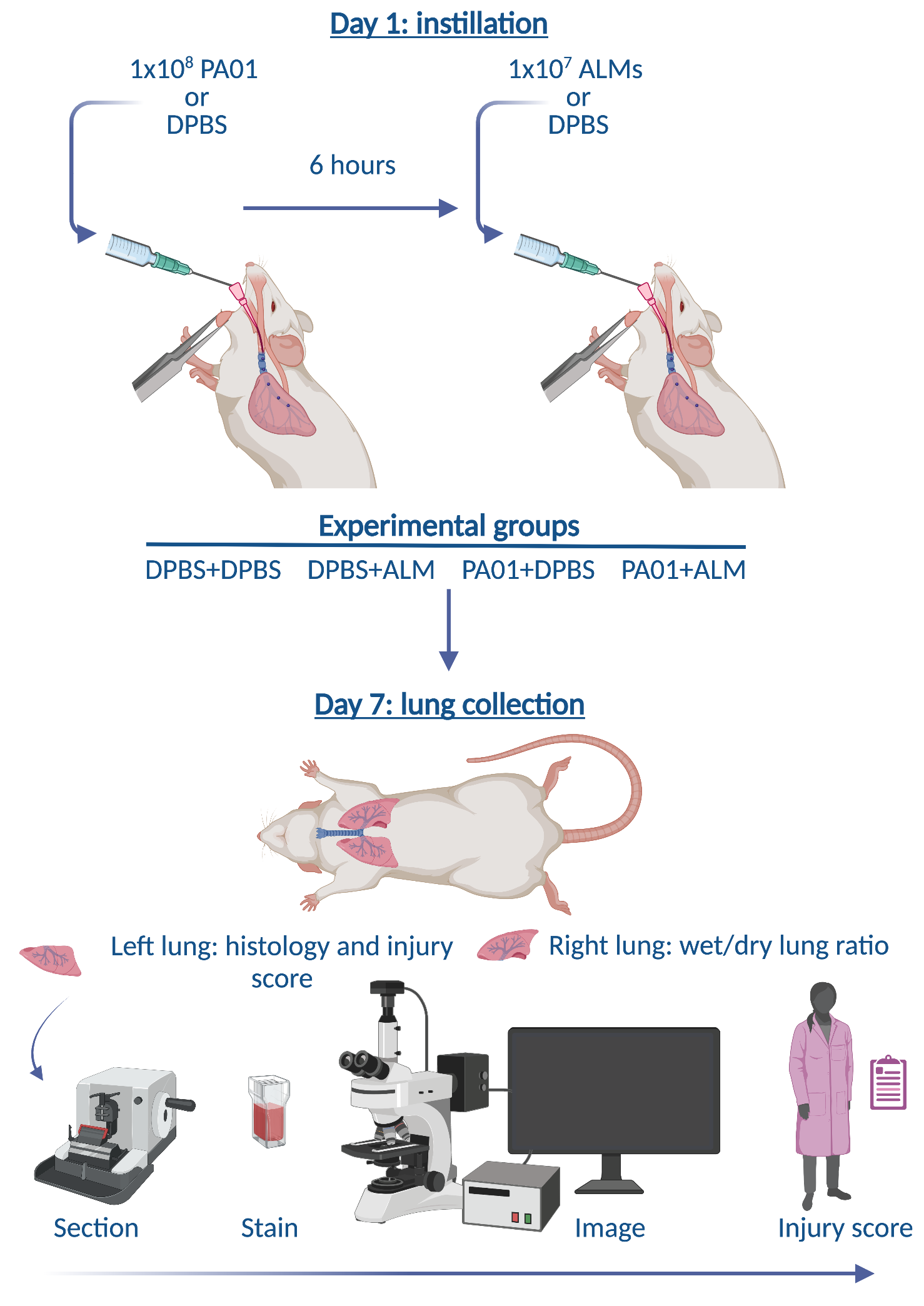

Supplement: Supplementary file 3 — Fig S3 [file JCMM-26-3046-s008.png]

**A**

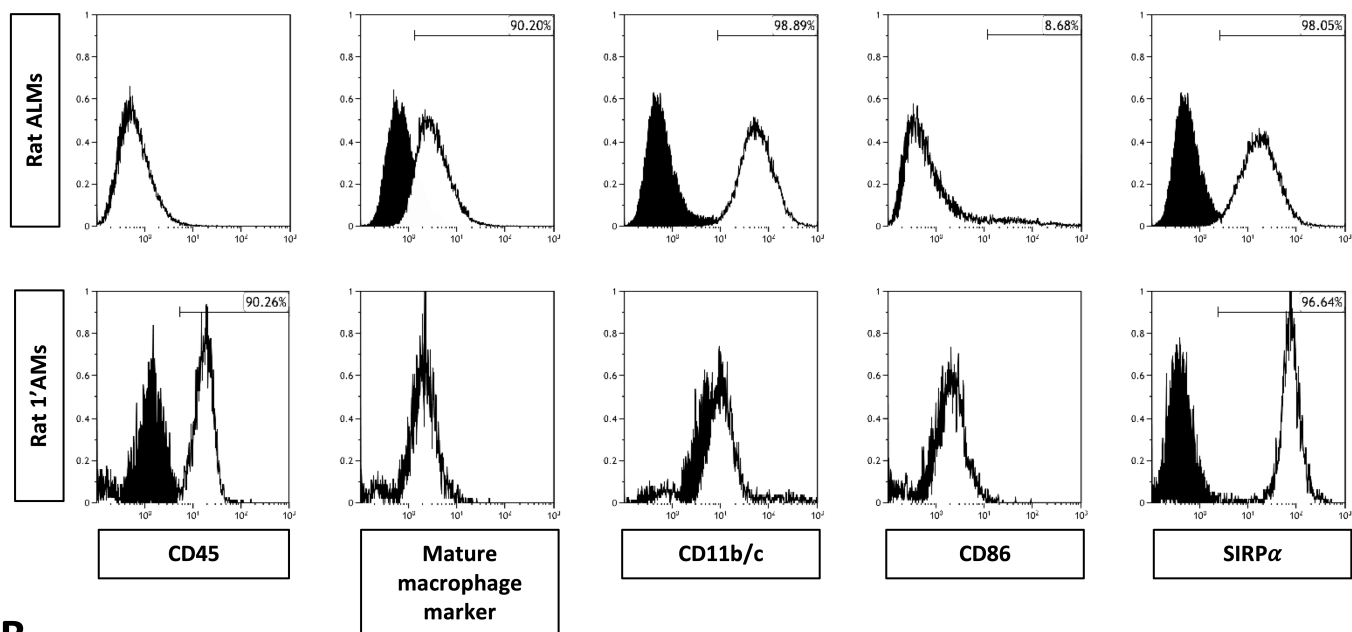

**B**

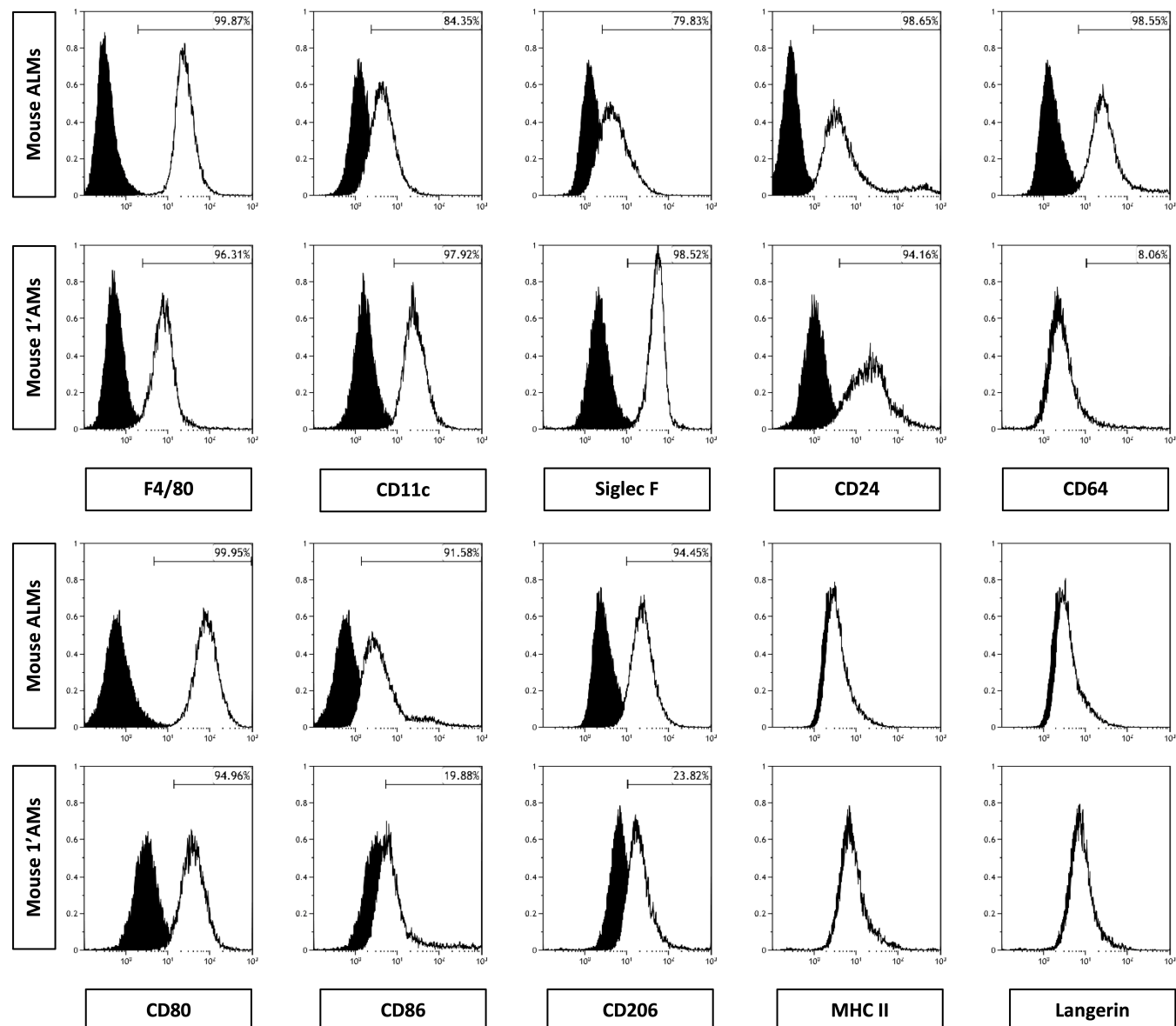

Supplement: Supplementary file 4 — Fig S4 [file JCMM-26-3046-s001.pdf]

**A**

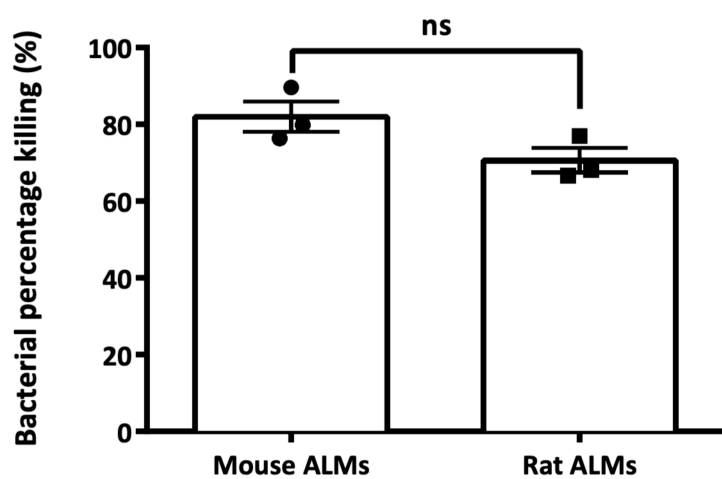

**B**

| Antibiotic survival status | Strain ID# |
|----------------------------|------------|
| ER                         | 288        |
| ER                         | 368        |
| ER                         | 510        |
| ER                         | 549        |
| ER                         | 558        |
| PR                         | 330        |
| PR                         | 342        |
| PR                         | 375        |
| PR                         | 505        |
| PR                         | 580        |

**C**

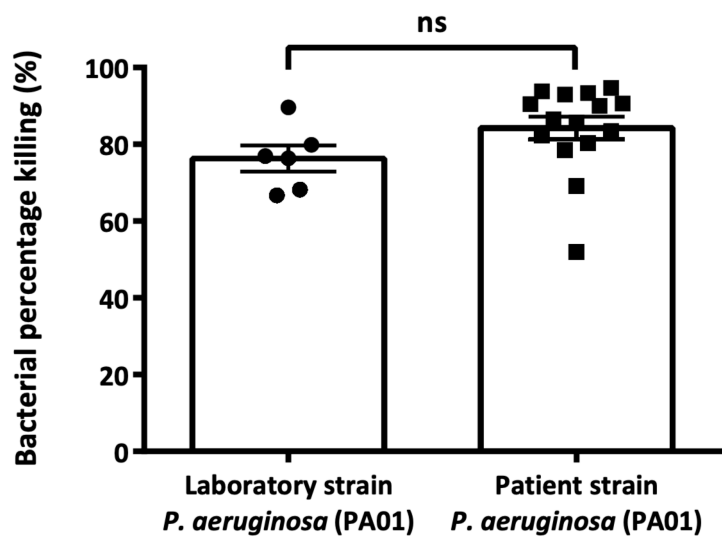

Supplement: Supplementary file 5 — Fig S5 [file JCMM-26-3046-s005.pdf]

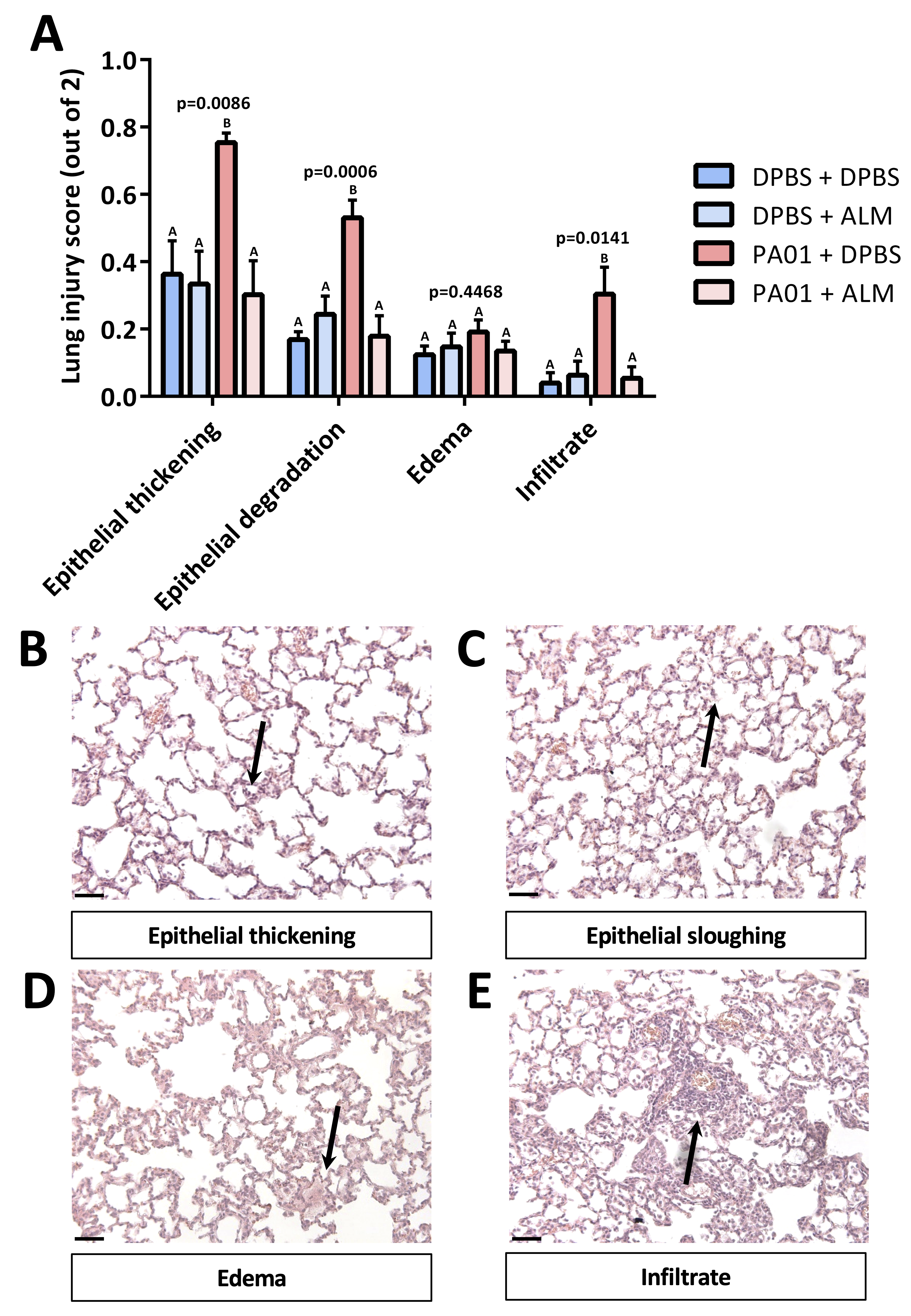

Supplement: Supplementary file 6 — Fig S6 [file JCMM-26-3046-s009.png]
