# Supplementary material for: Therapeutic stem cell‐derived alveolar‐like macrophages display bactericidal effects and resolve Pseudomonas aeruginosa‐induced lung injury
Source: J Cell Mol Med. 2022 Apr 20;26(10):3046–59. doi: 10.1111/jcmm.17324 (PMC9097833; doi:10.1111/jcmm.17324)
Supplement: Supplementary file 7 — Table S1 [file JCMM-26-3046-s006.docx]

**Supplementary Table 1. Details of reagents used for cell culture, gentamicin protection assays and animal experiments**

| **Reagent** | **Company** | **Product number** |
| --- | --- | --- |
| DMEM/F12 | Life Technologies | 11330-032 |
| FBS | Life Technologies | 12483-020 |
| GM-CSF | R&D Systems | 415-ML/CF |
| M-CSF | R&D Systems | 416-ML/CF |
| 1% (v/v) penicillin-streptomycin | Life Technologies | 15140122 |
| ReLeSR | StemCell Technologies | 05873 |
| Euthanyl (pentobarbitol) | Bimed-MTC | DIN 00141704 |
| DPBS | Life Technologies | 14040216 |
| HBSS | Life Technologies | 14025092 |
| HEPES | Sigma | H4034 |
| FluoroBrite DMEM | Fisher Scientific | A1896701 |
| Carbenicillin | Fisher Scientific | 10177012 |
| Gentamicin | Gibco | 15750060 |
| MEM | Gibco | 11095080 |
| Cryopreservation beads | Pro-Lab Diagnostics MicrobankTM | PL.170 |
| Lennox L Broth | Invitrogen | 12780052 |
| Saponin | Sigma | 47036-50G-F |
| DNAse I | Worthington | LSO2007 |
| LB agar | Invitrogen | 30391-023 |
| Ketamine | Vetoquinol | 440894 |
| Xylazine | Rompun Bayer | N/A |
| Isoflurane | Fresenius Kabi | CP0406V2 |
| PFA | Electron Microscopy Sciences | 15713 |
