# Supplementary material for: Therapeutic stem cell‐derived alveolar‐like macrophages display bactericidal effects and resolve Pseudomonas aeruginosa‐induced lung injury
Source: J Cell Mol Med. 2022 Apr 20;26(10):3046–59. doi: 10.1111/jcmm.17324 (PMC9097833; doi:10.1111/jcmm.17324)
Supplement: Supplementary file 8 — Table S2 [file JCMM-26-3046-s003.docx]

**Supplementary Table 2. Details of antibodies and reagents used for flow cytometry and fluorescence-activated cell sorting experiments**

| **Antibody/Reagent** | **Target** | **Concentration** | | **Company** | | **Product number** |
| --- | --- | --- | --- | --- | --- | --- |
| CellTrace™ Far-Red Cell Proliferation | Mouse and rat | 1:1000 | | Invitrogen | | C34564 |
| Anti-CD32 | Rat | 1:100 | | BD Biosciences | | 550270 |
| Anti-CD16/32 | Mouse | 1:100 | | BioLegend | | 101302 |
| Anti-TLR2 | Rat | 1:100 | | Bioss | | BS-1019R-PE |
| Anti-TLR4 | Rat | 1:100 | | Invitrogen | | PA5-23125 |
| Anti-TLR5 | Rat | 1:100 | | Invitrogen | | PA1-41139 |
| Anti-TLR2 | Mouse | 1:100 | | Invitrogen | | 50-9021-80 |
| Anti-TLR4 | Mouse | 1:100 | | Invitrogen | | 62-9041-80 |
| Anti-TLR5 | Mouse | 1:100 | | BioLegend | | 148103 |
| Anti-CD45 | Rat | 1:100 | | eBioscience | | 48-0461-80 |
| Anti-CD11b/c | Rat | 1:100 | | eBioscience | | 46-0110-80 |
| Anti-SIRPα | Rat | 1:100 | | eBioscience | | 17-1720-80 |
| Mature macrophage marker | Rat | 1:100 | | eBioscience | | 12-0660-82 |
| Anti-Ki67 | Mouse and rat | 1:100 | | Invitrogen | | 48-5698-80 |
| VivaFix | Mouse and rat | 1:1500 | | BioRad | | 135-1112 |
| Anti-CD80 | Mouse | 1:100 | | Invitrogen | | 25-0801-82 |
| Anti-CD86 | Mouse | 1:100 | | Invitrogen | | 25-0862-82 |
| Anti-CD206 | Mouse | 1:100 | | Invitrogen | | 48-2061-82 |
| Anti-Arg1 | Mouse | | 1:100 | Invitrogen | 25-3697-82 | |
| Anti-iNOS | Mouse and rat | | 1:100 | Invitrogen | 48-5920-82 | |
| Anti-Siglec F | Mouse | 1:100 | | Invitrogen | | 62-17920-80 |
| Anti-CD11b | Mouse | 1:100 | | Invitrogen | | 48-0112-80 |
| Anti-GR-1 | Mouse and rat | 1:100 | | Invitrogen | | 12-5931-81 |
| Anti-CD80 | Rat | 1:100 | | Invitrogen | | 12-0800-82 |
| Anti-CD86 | Rat | 1:100 | | Invitrogen | | 12-0860-83 |
| Anti-CD206 | Rat | 1:100 | | Bioss | | BS-47727R-PE |
| Anti-Arg1 | Rat | 1:100 | | Bioss | | BS-8585R-PE |
