# Supplementary material for: Therapeutic stem cell‐derived alveolar‐like macrophages display bactericidal effects and resolve Pseudomonas aeruginosa‐induced lung injury
Source: J Cell Mol Med. 2022 Apr 20;26(10):3046–59. doi: 10.1111/jcmm.17324 (PMC9097833; doi:10.1111/jcmm.17324)
Supplement: Supplementary file 9 — Figure Lagends [file JCMM-26-3046-s004.docx]

**Fig S1:** **Schematic representation of experimental design to determine in vivo internalization of bacteria by alveolar‐like macrophages.** To assess in vivo internalization of bacteria by mouse DsRed^+^ and rat CellTrace™^+^ ALMs, live GFP expressing bacteria were instilled in mice and rats (1x10^7^ and 1x10^8^ bacteria, respectively). 30 min later ALMs were instilled (mice; 1x10^6^ ALMs, rats; 1x10^7^ ALMs). Three hours post‐instillation BALF was collected. Flow cytometry was performed on the BALF, gating for GFP (bacteria) and DsRed^+^ ALMs or CellTrace™^+^ ALMs. Mouse GFP^+^DsRed^+^ and rat GFP^+^CellTrace™^+^ cells were considered ALMs that had internalized bacteria. Internalization was confirmed by creating cytospots and ensuring co‐localization of DsRed or CellTrace™ and GFP staining using confocal microscopy

**Fig S2:** **Schematic representation of experimental design to determine in vivo killing capacity of bacteria by alveolar‐like macrophages.** To assess rat ALMs ability to kill bacteria over 24 h in vivo and alter bacterial load within the lung, 1x10^8^ PA01 was instilled followed by 1x10^7^ CellTrace™^+^ ALMs 30 min later. BALF was collected at T0 (3 h) or T24 (27 h) post‐PA01 instillation. BALF was centrifuged and the supernatant (containing planktonic bacteria) was plated onto LB‐Agar plates and colony forming units were counted to assess overall bacterial load within the lung. To assess the killing capacity of ALMs and primary BALF cells, CellTrace™^+^ ALMs were separated from CellTrace™^‐^ primary BALF cells by Fluorescence‐Activated Cell Sorting. Sorted cells were centrifuged onto cytospots or lysed and plated onto LB‐Agar plates and colony forming units were counted. Bacterial killing capacity was calculated by comparing the T0 and T24 time points

**Fig S3:** **Schematic representation of experimental design to determine the role of alveolar‐like macrophages in P. aeruginosa‐induced lung injury in vivo.** A lung injury model was established by intratracheally instilling 1x10^8^ PA01 into rats, with controls receiving a vehicle (DPBS). Six hours after either PA01 or DPBS instillation both groups were instilled with either 1x10^7^ ALMs or a DPBS control resulting in 4 experimental groups DPBS + DPBS, DPBS + ALM, PA01 + DPBS, PA01 + ALM. At Day 7 the right lung was collected to determine the wet/dry weight ratio and the left lung was pressure fixed, sectioned and stained with hematoxylin and eosin. From each rat, 60 images were randomly taken in a grid‐like fashion on a light microscope. An injury score was assigned to each image based on the presence and severity of epithelial thickening, epithelial sloughing, oedema and infiltrate

**Fig S4:** **Characterization of cell surface markers expressed by mouse and rat primary alveolar macrophages and alveolar‐like macrophages.** (A) Rat ALMs express SIRPα, CD11b/c, mature macrophage marker, but do not express the myeloid marker CD45, and CD86, when cultured in vitro whereas rat 1’AMs express CD45 and CD86 (n = 4 separate isolations). Black histograms; unstained control cells, white histograms; stained cells. All histograms are representative of n = 4‐5 experiments. (B) As previously published in tabular format in [17](file:///Z:\journals\W3G\TEST_ELD\JQAVendorTool_2.25.6\out\JCMM_17324\Preview\JCMM_17324\wol.html#jcmm17324-bib-0017), mouse 1’AM and ALMs expression patterns

**Fig S5:** **Comparisons between mouse and rat alveolar‐like macrophages of their bactericidal killing capacity of both laboratory and clinical strains of P. Aeruginosa.** (A) There was no difference in the bactericidal killing capacity of the laboratory strain of P.A. between mouse and rat ALMs. (B) List of P.A. clinical isolates and their antibiotic survival status. (C) There was no difference in the bactericidal killing capacity of the laboratory strain of P.A. compared to patient derived clinical stains that are either tobramycin sensitive and are eradicated by antibiotics (ER) or are resistant to tobramycin and persist with antibiotic treatment (PR) by mouse and rat alveolar‐like macrophages combined

**Fig S6:** **Characterization of lung injury score in P. aeruginosa‐induced lung injury model.** (A) Further breakdown of the injury score observed in the P.A.‐induced lung injury model determined that the greater injury score in the PA01 + DPBS group was due to an increase in epithelial thickening, epithelial sloughing and infiltrate, but not oedema. Data are mean ± SEM, n = 3‐4 rats per experimental group. Experimental groups with different letters (above the bar) are significantly different from each other. (B), (C), (D) and (E) Representative histological images of epithelial thickening, epithelial sloughing, oedema and infiltrate, respectively. Images were taken at x200 magnification. Black arrows indicate injury. Scale bars are 50 μm
